# Surgery of T1/2 N0 M0 glottic cancer results in a better laryngeal preservation time compared to radiotherapy in a large German patient cohort

**DOI:** 10.1007/s00405-025-09460-3

**Published:** 2025-06-14

**Authors:** Manuel Christoph Ketterer, Thomas K. Hoffmann, Simon Laban, Alexander Berghaus, Martin Canis, Christian Jacobi, Jens Peter Klussmann, Wendelin Foeringer, Roland Laszig, Jens Pfeiffer, Henning Bier, Andreas Knopf

**Affiliations:** 1 Department of Otorhinolaryngology – Head and Neck Surgery, University Medical Centre Freiburg, Faculty of Medicine, University of Freiburg, Killianstraße 5, 79106 Freiburg, Germany; 2 Department of Otorhinolaryngology, Head and Neck Surgery, Ulm University Medical Center, Frauensteige 12, 89075 Ulm, Germany; 3 Department of Oto-Rhino-Laryngology, Head and Neck Surgery, Grosshadern Campus Ludwig-Maximilian-University Munich, Marchioninistraße 15, 81377 Munich, Germany; 4 Department of Otorhinolaryngology, Head and Neck Surgery, University of Giessen, Klinikstrasse 33, 35392 Giessen, Germany; 5 Otorhinolaryngology/Head and Neck Surgery, Klinikum Rechts Der Isar, Technical University Munich, Ismaninger Str. 22, 81675 München, Germany; 6 Department of Otorhinolaryngology, Head and Neck Surgery, University of Cologne, Medical Faculty, Cologne, Germany

**Keywords:** Glottic laryngeal carcinoma, Overall survival, Transoral laser surgery, Open partial vertical laryngectomy, Surgical management, Head and neck cancer, radiotherapy

## Abstract

**Objective:**

This retrospective study examined overall survival (OS), recurrence free survival (RFS), and laryngeal preservation time in a large cohort of 663 patients with T1/2 N0 M0 glottic cancer after transoral laser or open surgery vs radiotherapy.

**Methods:**

A total of 595 surgically treated patients and 68 individuals after definitive radio(chemo)therapy (R (C)–T) were studied. Patient characteristics including sociological, surgical, and pathological data, OS and RFS as well as laryngeal preservation time were recorded and compared between various groups/cohorts.

**Results:**

There were no significant differences in OS and RFS between surgically treated and conservatively treated patients. However, laryngeal preservation time was significantly higher in surgically treated patients (*p* < 0.001) (mean: 138.3 ± 2.2 months, versus 102.8 ± 7.6 months) than those under conservative treatment. The surgical treatment method (transoral vs. open partial resection) did not influence OS or RFS. Additionally, the rate of transoral vs. open surgery did not change over a decade. T2-stage patients showed significantly lower RFS than T1-stage patients. Initial R status significantly influenced OS and tumor recurrence.

**Conclusion:**

The findings of this study exhibited a significantly longer laryngeal preservation time in patients with T1/2 N0 M0 glottic cancer treated surgically than in those treated with radiotherapy. No significant differences in OS or RFS were observed between open partial laryngectomy and transoral laser surgery. The R status had a significant impact on OS and RFS, with OS being significantly associated with an R0 status, regardless of T status or the surgical approach (open versus transoral). Laryngeal preservation surgery is recommended as a central therapeutic strategy for T1/2 N0 M0 glottic cancer because it has a higher laryngeal preservation rate than the conservative treatment. Given the high recurrence rate (18.5%) and the necessity of laryngo- (pharyng) ectomy in a substantial proportion of recurrent patients (7.7%), the choice of initial therapeutic approach is critical.

## Introduction

Laryngeal squamous cell carcinoma (LSCC) is one of the most common types of head and neck cancer [1, 2]. Its mortality and incidence rates have decreased in Europe and the United States over the last 20 years, most likely due to socioeconomic factors and therapeutic advancements [3]. T1 and T2 N0 glottic LSCC (early LSCC) are common types with a favorable prognosis, and five-year survival rates of 90% and 80%, respectively [4, 5]. This is largely due to early detection and limited lymphatic drainage [4, 5]. Preserving the larynx and its function is a key element in the treatment of early-stage glottic LSCC. To achieve this, laryngeal preservation surgery (LPreS) and radiotherapy (RT) are used and they normally produce the same results in terms of traditional outcomes, such as overall survival (OS) and functional outcomes [1, 6].

Regarding LPreS, Strong and Jako [7] first described the use of CO_2_ laser for transoral laryngeal surgery in 1972, establishing an alternative to open laryngeal surgery, which had been the standard approach. Some previous studies have confirmed comparable oncological outcomes between laser surgery and open partial laryngectomy for T1 glottic cancer [8–10]. However, open partial laryngectomy is associated with higher trauma than transoral surgery or RT. The use of open laryngeal surgery has decreased [1] due to the increasing use of radio(chemo)therapy (R(C)T) [11, 12] and improved transoral laser surgery techniques [13]. Additionally, recent case series [14, 15] reporting on transoral robotic approaches have shown survival rates comparable to those of transoral laser surgery in patients with T1 glottic cancer. However, not all patients with glottic LSCC can be treated via transoral approaches owing to the reduction in mobility of the cervical spine with age, improved dental status, and tumor localization, especially involvement of the potential anterior commissure [16, 17]. To date, no randomized prospective clinical trials have evaluated and compared both LPreS approaches (transoral laser surgery and open laryngeal surgery), due to patient bias and ethical reasons. Additionally, there are no published studies on the survival and recurrence rates of T1 and T2 glottic carcinoma comparing surgical with conservative treatment or transoral laser with open partial laryngectomy [13]. Brumund et al. [18] published a study including 270 patients in which they compared laryngeal preservation, OS, and local control. Follow-up studies [18–21] included fewer patients. Additionally, these studies included patients with T1, T2, and T3 glottic carcinoma. However, open partial laryngectomy is uncommon in T3 carcinoma surgery.

This study aimed to compare OS and recurrence free survival (RFS) and also the follow-up period in terms of laryngeal preservation between conservatively treated patients with R(C)T and surgically treated patients with LPreS. Additionally, this study investigated whether various surgical modalities, such as open surgical treatment via partial laryngectomy, influence OS and RFS differently from transoral laser surgery. Furthermore, this study aimed to analyze differences in OS and RFS, R status, adjuvant therapy, and recurrence between patients with T1 and T2 N0M0 surgically treated glottic cancer.

## Methods

### Study cohort

This retrospective multicenter study was conducted at five university hospital centers (including four comprehensive cancer centers) in Germany (Klinikum rechts der Isar, Technical University Munich, Ulm University Medical Center, Ludwig Maximilians University of Munich, University of Giessen, and University Medical Center Freiburg).

This study included 595 surgically treated patients with pT1/2 c/pN0 cM0 glottic cancer and 68 patients conservatively treated with cT1/2 cN0 cM0 glottic cancer treated with R(C)T between January 2004 and December 2014. Of the 68 conservatively treated patients, 47 underwent RT, and 19 underwent RCT. Data were collected on parameters including age, sex, tumor characteristics (size, stage, histopathology, tumor location, TNM status, grading, treatment modalities, R status, and treatment), follow-up data, clinical parameters, and survival data (recurrence and death/loss to follow-up). Patients with carcinoma *in situ* or dysplasia and other histological subtypes, such as adenocarcinoma, and those with missing data, incomplete staging, those who refused or did not finish treatment, and those aged < 18 were excluded from the study. Tumors were staged according to the guidelines given in the Union International Control Cancer (UICC) staging system, version 7. Surgeries were performed via open partial laryngectomy when visibility during laryngoscopy was inadequate, particularly due to anterior commissure involvement.

### Statistical analysis

Statistical analysis was performed using SPSS (IBM Corp. Released 2015. IBM SPSS Statistics for Windows, Version 24.0, Armonk, NY, USA). Parameters were compared between the two groups using the Wilcoxon test. A *p*-value < 0.05 indicated statistical significance. Survival analyses were performed using the Kaplan–Meier method, and univariate analysis was performed using the log-rank test. Comparisons between groups with different T status were performed. The primary predictor variables were T status and surgical treatment, and the variables of interest were sex, age, year of treatment, stage, and adjuvant therapy. OS, the primary outcome variable, was defined as the time from the date of diagnosis to death, using time-to-event analysis. RFS, the secondary outcome variable, was defined as the time from the date of diagnosis to the date of recurrence. Time trends were analyzed using linear regression. Laryngeal preservation time was defined as a time-to-event, with L(P)E as the defining event.

### Ethical considerations

This study was approved by local ethics committees of the participating institutions.

## Results

A total of 595 surgically treated and 68 conservatively treated (R(C)T) T1 or T2 N0M0 glottic cancer patients were included in the analysis. Out of the 595 surgically treated patients, 470 were diagnosed with T1 N0M0 glottic cancer and 125 were diagnosed with T2 N0M0 glottic cancer. Out of the 68 conservatively treated patients, 36 were diagnosed with T1 N0M0 glottic cancer and 32 were diagnosed with T2 N0M0 glottic cancer (Table 1). The 663 patients included in this study comprised 607 males and 56 females.

**Table 1:** Study cohort of 595 surgically treated c/pT1 c/pN0 cM0 glottic cancer patients and 68 conservatively treated patients following radio(chemo)therapy

	Laryngeal preservation Surgery (LPreS)		Radio(chemo) therapy (R(C)T)	*p*
	Transoral	Partial laryng		
T1 (*n*)	416	54	36	/
T2 (*n*)	84	41	32	/
Age (years ± SD)	63.8 ± 10.1		65.5 ± 11.8	0.053
OS (months ± SD)	114.1 ± 3.5	111.2 ± 7.4	122.7 ± 8.1	0.8
RFS (months ± SD)	120.1 ± 3.4	117.4 ± 7.3	91.9 ± 8.1	0.1

**Fig. 1:**
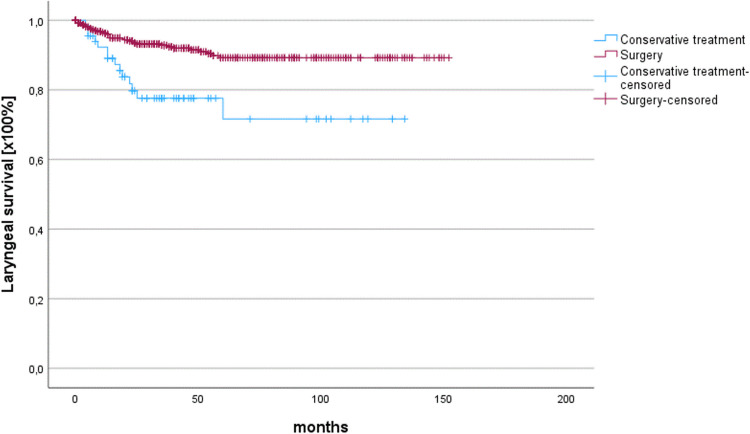
Laryngeal preservation time calculated as time to reach laryng- (opharyng)ectomy is significantly better in initially surgically treated patients with laryngeal preserving surgery than in conservatively treated patients following radio(chemo)therapy (*p* < 0.001)

There were no significant differences in OS and RFS between LPreS and R(C)T in patients with T1/2 N0M0 glottic cancer (Table 1). Our previous study revealed that about 20% of our patients experienced local disease recurrence and underwent laryng- (opharyn) gectomy (L(P)E) [22]. Generally, the estimated laryngeal preservation time (avoiding L(P)E) has a major clinical impact. Therefore, the laryngeal preservation time was compared between LPreS and primary R(C)T using Kaplan–Meier analysis up to L(P)E. The laryngeal preservation time was significantly better in surgically treated patients than in those treated with primary R(C)T (*p* < 0.001) (Fig. 1). Patients who underwent LPreS had a mean laryngeal preservation time of 138.3 ± 2.2 months, whereas conservatively treated patients following R(C)T had a mean laryngeal preservation time of only 102.8 ± 7.6 months.

**Fig. 2:**
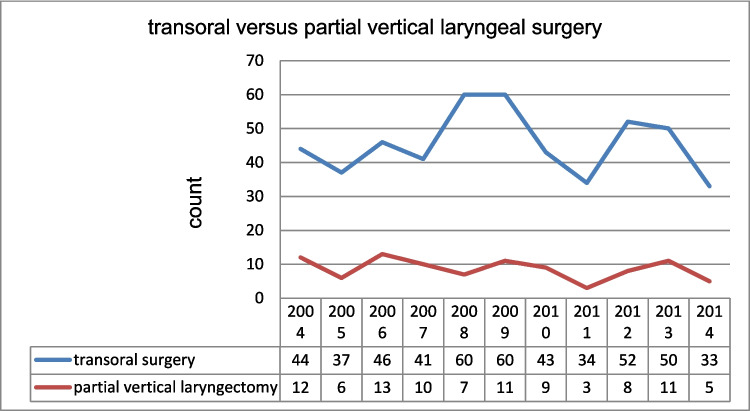
Time trends for transoral surgery versus open partial laryngeal surgery showing no significant time trends over 10 years (*p* = 0.66)

Subsequently, our analysis focused on the various surgical concepts of LPreS (Fig. 2). No significant time trends for transoral laser surgery and open partial laryngectomy were observed over the 10 years (*p* = 0.675), indicating that both surgical concepts had remaining evidence and indications. Additionally, the number of conservatively treated patients was stable over time. Anatomical condition and T status are key in determining whether LPreS is performed transorally or through open partial laryngectomy. Therefore, the two surgical approaches were examined for differences (Table 2). The results showed that open laryngeal surgery (open partial laryngectomy) was significantly more frequently performed in T2-stage than in T1-staged patients (*p* < 0.018). The mean ages of the T1- and T2-stage patients were 64 and 62 years, respectively, and no significant difference in age or number of males and females was observed between the two groups. Comparisons between T1- and T2-stage patients using the Chi-square test for cohorts > 20 and the exact Fisher test for smaller study groups showed significant differences in grading with more a pronounced occurrence of G1 status in T1 cancer with transoral approaches (*p* < 0.001).

**Table 2:** Numbers of patients in the study cohort undergoing transoral surgery and open partial laryngectomy under T1 vs T2 N0M0 (*indicates that data of seven patients are missing)

	T1		T2		*p*
Surgical approach	transoral	partial laryng	transoral	partial laryng	
*n*	416	54	84	41	0.018
age (years)	64 ± 10		62 ± 9		0.294
sex, n					0.489
Male	378	53	76	38	
Female	38	1	8	3	
G *					< 0.001
1	98	9	5	3	
2	289	41	57	29	
3	24	4	20	9	
R					0.001
0	338 81%	52 96%	65 77%	39 95%	
1	16 4%	0 0%	9 11%	0 0%	
x	62 15%	2 4%	10 12%	2 5%	
adjuvant RT					0.378
yes	5 1%	0	12 14%	5 12%	
none	411 99%	54 100%	72 86%	36 88%	

**Fig. 3:**
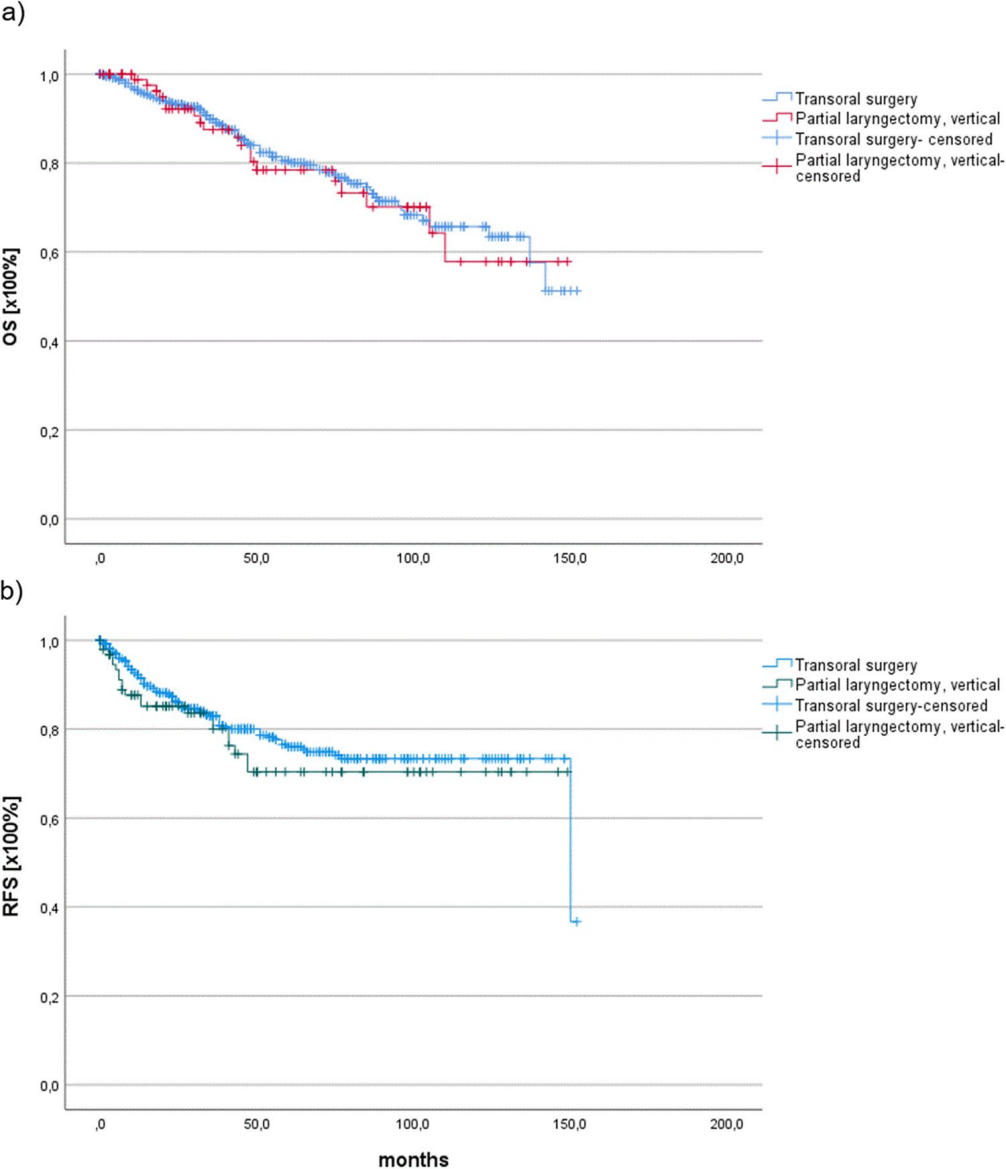
Overall survival (OS) analysis (**a**) and recurrence free survival (**b**) under transoral surgery versus open partial laryngectomy showing no significant survival differences in T1/2 N0M0 glottic carcinoma patients (OS: *p* = 0.8) (RFS: *p* = 0.355)

There was a highly significant difference in the R status between T1- and T2-stage patients. Transorally resected glottic cancer patients had a higher frequency of R1 status in both T2 (11%) and T1 LSCC (4%). Additionally, in T1- and T2-stage patients, transoral laser surgery led to a significantly more frequent pathological Rx-status, which might be attributed to the surgeon’s piecemeal technique, as previously described by Backes et al. [23]. As there were no significant differences in the proportion of adjuvant-treated patients (RT) between T1- and T2-stage patients (*p* = 0.378), the Rx-status was interpreted as R0, indicating no adjuvant therapy escalation. A comparison of T1/2 N0M0-stage patients regarding their surgical management (transoral laser surgery vs. open partial laryngectomy) showed no significant differences in OS (*p* = 0.8) or RFS (*p* = 0.355) (Fig. 3a and b and Table 1). Additionally, by separately calculating OS and RFS for T1 N0M0- and T2 N0M0-stage patients, no significant differences were observed between transoral laser surgery and open partial laryngectomy. Furthermore, there were no significant differences in mean OS between T1 (118.65 ± 3.2 months) and T2 N0M0 glottic cancer patients (105.56 ± 6.96 months) (*p* = 0.119). However, a significant decrease in mean RFS was observed in T2-stage carcinoma patients (104.4 ± 6.8 months) relative to T1-stage carcinoma patients (120.75 ± 3.1 months) (*p* = 0.004).

**Fig. 4:**
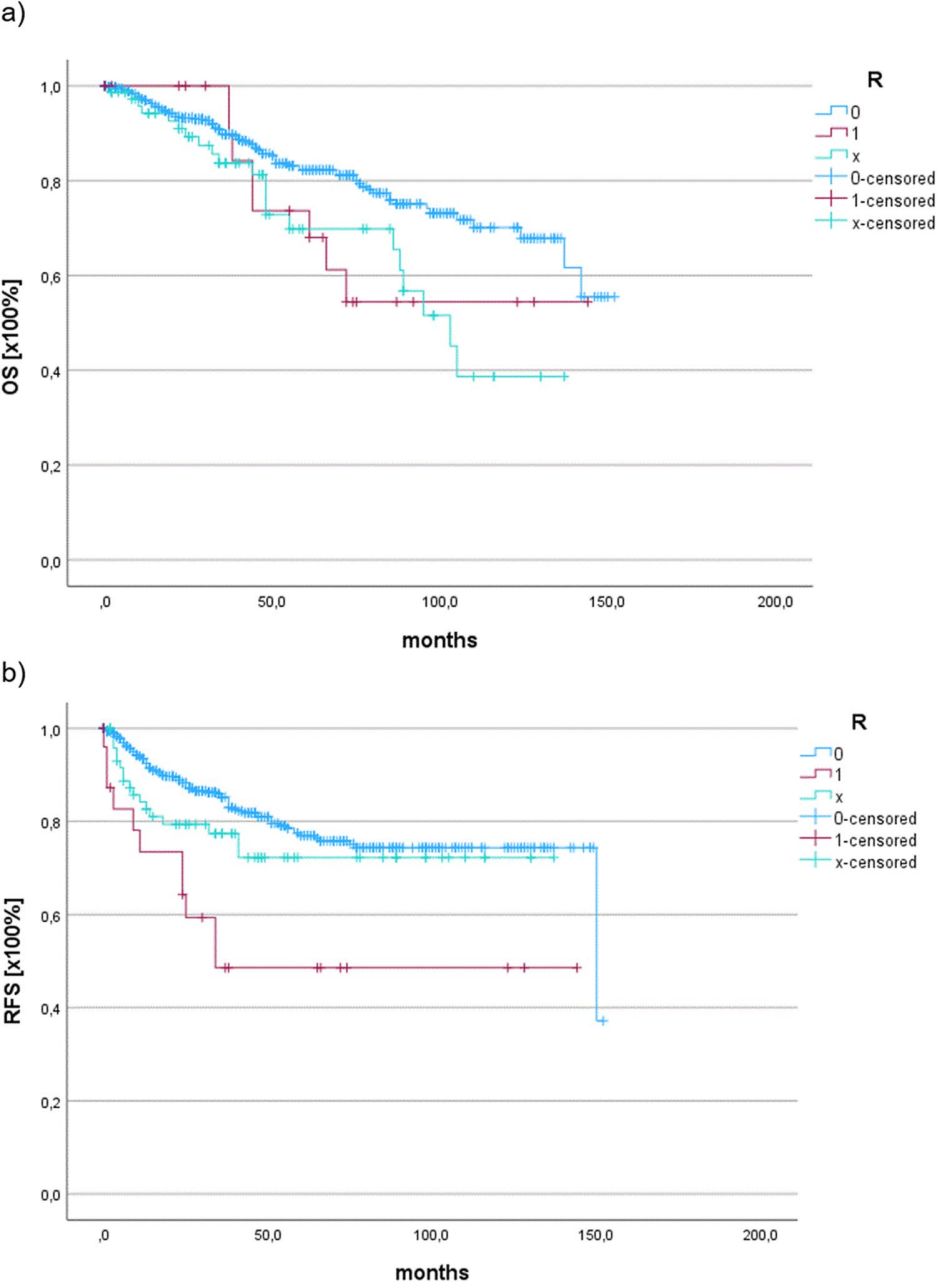
Significantly lower OS (**a**) and RFS (**b**) for R1 and Rx than for R0 in glottic T1/2 N0M0 laryngeal cancer patients (*p* = 0.013)

A significant decrease in OS was observed among R1-, Rx-, and R0-dissected patients (*p* = 0.013) (Fig. 4a). Additionally, R1-dissected patients exhibited a significantly lower RFS than Rx- and R0-dissected patients (*p* < 0.001) (Fig. 4b). Cox regression analysis confirmed that the R status significantly influenced OS (*p* = 0.034) and RFS (*p* = 0.046). A comparable RFS was observed among the 22 patients who received adjuvant treatment with radiotherapy and those who did not (p = 0.934). The non-adjuvant-treated group showed a longer OS (mean: 117.5 ± 2.99 months) than the adjuvant-treated group (mean: 85.15 ± 14.8 months), though this difference was not statistically significant (p = 0.050).

One hundred and ten (18.5%) patients experienced local tumor recurrence, and 46 patients (7.7%) underwent L(P)E due to tumor recurrence. Thirty one patients underwent further transoral tumor resection. Forty three recurrence patients were treated with R(C)T, 11 of them in palliative situation. The dependence of OS in tumor recurrence on initial R status was examined using the Chi-square test. The results showed a significantly higher OS in initial R0-dissected patients (mean: 92.23 ± 6.7 months) than in R1-dissected patients (mean: 70.4 ± 7.1 months) and Rx-dissected patients (mean: 52.96 ± 8.7 months) (*p* = 0.008). Neither initial surgical treatment (transoral vs. open partial laryngectomy) (*p* = 0.719) nor initial T status (T1 vs. T2) (*p* = 0.781) had a significant influence on OS in tumor recurrence.

## Discussion

Given the favorable prognosis of T1/2 N0M0 glottic cancer and the fact that LPreS and R(C)T have similar outcomes in terms of OS and RFS [22] and similar functional outcomes, the question arises: What is the optimal therapeutic strategy for laryngeal preservation in T1/2 N0M0 glottic cancer?

The findings of this study demonstrated that laryngeal preservation in T1/2 N0M0 glottic cancer was significantly better in surgically treated patients than in those treated with primary R(C)T. We compared 595 surgically treated patients with 68 conservatively treated patients (R(C)T). Although this difference in case numbers may be a limitation of the study, it did not affect the findings, as OS/RFS and major clinical parameters of the study cohorts did not significantly differ between LPreS and R(C)T.

Kaplan–Meier analysis up to the time of LPE revealed significantly better laryngeal survival time for LPreS than for primary R(C)T). RT is an established conservative, alternative treatment method, and previous studies reported comparable local control and OS between transoral surgery and RT for T1 and T2 glottic laryngeal SCC [6, 24–26]. However, Abdurehim et al. [26] reported higher larynx preservation in tumor recurrence after initial laser surgery than after initial RT for T1a glottic laryngeal cancer.

In this study, the LPreS cohort was analyzed to determine the effects of LPres on OS, RFS, and recurrence status. A comparison of the surgical modalities showed no significant positive or negative time trends between transoral laser surgery and open partial laryngectomy. These findings indicate that both surgical modalities have indications, as demonstrated by the stable number of procedures over a period of 10 years in five university hospital (four comprehensive cancer) centers in Germany. Additionally, there were no significant differences in OS and RFS between transoral surgery and open partial laryngectomy in T1/2 N0M0 glottic carcinoma patients. Previous studies reported that transoral laser surgery and open partial laryngectomy had comparable oncological outcomes [27–29]. However, partial laryngectomy is surgically associated with high morbidity, frequent need for a temporary tracheostomy [30, 31], and longer hospitalization times [32]. Previous studies recommended open partial laryngectomy for treating early-stage glottic cancer and open partial laryngectomy only in T1 N0M0 glottic cancer patients and in cases of transglottic LSCC in T2 glottic cancer, without impaired vocal cord movements [17, 33, 34]. As anatomical condition and T-status are key factors in deciding whether LPreS is performed transorally or through open partial laryngectomy, T1 N0M0 and T2 N0M0 glottic cancer patients were further compared. The results indicated that T2-stage patients were more frequently treated with open partial laryngectomy than T1-stage patients (*p* = 0.018). Brumund et al. [18] reported a significantly lower OS for T2 N0M0 tumors than for T1 N0M0 tumors. These findings are inconsistent with those of our study, in which the number of T2 tumors in our study cohort was lower, and therefore, a bias could not be ruled out. Brumund et al. demonstrated a significant influence of a high frequency of T-status and local failure on OS [18]. Additionally, they included not only T1- and T2-stage patients but also T3-stage patients, which made comparisons difficult. Although we did not observe the lower OS for T2 N0M0 than for T1 N0M0, as described by Brumund et al. [18], transoral resected T2 glottic cancer resulted in a higher frequency of R1 status (11%) than T1 status (4%).

In both T1- and T2-stage patients, transoral laser surgery resulted in a higher frequency of pathological Rx-status. This may be due to the surgeon’s piecemeal technique when performing the procedure [23]. As no significant differences were observed in the number of adjuvant-treated patients (RT) between the T1 and T2 stages, the Rx-status was interpreted as R0, indicating that both surgical methods were effective. However, a significant decrease in OS was observed in R1-dissected patients than in R0-dissected patients. The influence of the T-status, tumor location, and initial clinical staging on OS is highly disputed. Özkul et al. [17] reported a lower local control rate in patients with anterior commissure involvement who underwent frontolateral laryngectomy. Previous studies reported a relatively low success rate of RT in early-stage glottic cancer patients with anterior commissure involvement [35]. Possible reasons for this are the difficulty in staging of the anterior commissure and occult invasion of the thyroid cartilage. Celakovsky et al. [36] reported a disparity in at least one component of TNM staging in up to 32% of the patients included in the study. They concluded that the disparity in T-status in glottic cancer due to the upstaging from cT1 to pT4 significantly decreased OS and disease-specific survival.

Previous studies reported different local recurrence rates of T2-stage LSCC, with a limited discussion of the effect of anterior commissure involvement. Giovanni et al. [33] reported a local recurrence rate of 6% of T2 lesions. Fiorella et al. [34] reported a 14% higher local recurrence rate in T2-stage patients with anterior commissure involvement. In this study, all patients underwent laryngoscopy and biopsy before the decision to perform either transoral laser or open partial laryngeal surgery. As R1- and Rx-dissected patients showed a significant decrease in OS and RFS, the preoperative decision of either transoral or open partial surgery is highly important. Additionally, the initial R status significantly influenced OS.

In this study, the tumor recurrence rate was 18.5%, which is comparable to that reported in previous studies. Brumund et al. [18] reported a laryngeal preservation rate of 93.3% after 25 years of observation. These findings are consistent with those of our study, which showed that 7.7% of the patients (46 out of 595) required LPE due to tumor recurrence. A major limitation of this study was that surgical complications, laryngeal function, voice quality, and quality of life could not be examined due to the retrospective nature of the study. Therefore, further studies are needed to investigate health-related quality of life using psychometric tests in addition to survival and recurrence rates. Another limitation is the underlying differences in the patient groups that underwent LPreS and conservative treatment. Additionally, some of the conservatively treated patients underwent not only RT but also RCT, which is not recommended by the German laryngeal cancer guideline [37]. Further studies are needed to investigate the effect of anterior commissure and to examine T2 N0M0 glottic LSCC at various sites and their surgical outcomes.

## Conclusion

This study showed no significant differences in OS and RFS between T1- and T2-staged patients in one of the largest European cohorts of T1/2 N0M0 glottic cancer patients published to date. However, in T1/2 N0M0 glottic cancer, laryngeal preservation was a central aspect of therapy, and surgically treated patients showed significantly better laryngeal preservation time than conservatively treated patients. Regarding surgical modalities, open partial laryngectomy and transoral laser surgery had comparable OS and RFS. The choice between transoral and open laryngeal surgery is based on the localization and morphology of the carcinoma. However, surgical approaches have comparable success rates. The R status significantly negatively influenced OS and RFS, indicating that OS is significantly dependent on achieving a complete (R0) resection, independent of the T-status and open versus transoral surgical management. Owing to recurrence rates of 18.5% and the necessity of L(P)E in 7.7% of patience, we recommend that LPreS should have an additional dose available for possible recurrence irradiation.

## Data Availability

The data that support the findings of this study are available on request from the corresponding authors.

## Abbreviations

*LPreS*: Laryngeal preserving surgery
*R (C) T*: Radio (chemo) therapy
*RFS*: Recurrence free survival
*LSCC*: Laryngeal squamous cell carcinoma
*OS*: Overall survival
*L(P)E*: Laryng(opharyng)ectomy

## References

[1] Wiegand S (2016) Evidence-based review of laryngeal cancer surgery. Laryngorhinootologie 95(Suppl 1):S192-216 27128401 10.1055/s-0041-108962

[2] Krebs in Deutschland 2009/2010 9 (2013) Ausgabe. Robert Koch-Institut (Hrsg.) und die gesellschaft der epidemiologischen krebsregister in Deutschland e.V. (Hrsg.). Berlin

[3] Chatenoud L, Garavello W, Pagan E, Bertuccio P, Gallus S, La Vecchia C, Negri E, Bosetti C (2015) Laryngeal cancer mortality trends in European countries. Int J Cancer. 10.1002/ijc.29833 26335030 10.1002/ijc.29833

[4] Dirix P, Lambrecht M, Nuyts S (2010) Radiotherapy for laryngeal squamous cell carcinoma: current standards. Expert Rev Anticancer Ther 10:1461–1469 20836681 10.1586/era.10.110

[5] Chen JJ, Stessin A, Christos P, Wernicke AG, Nori D, Parashar B (2015) Differences in survival outcome between stage I and stage II glottic cancer: a SEER-based analysis. Laryngoscope 125:2093–2098 26109043 10.1002/lary.25338

[6] Feng Y, Wang B, Wen S (2011) Laser surgery versus radiotherapy for T1–T2N0 glottic cancer: a meta-analysis. ORL J Otorhinolaryngol Relat Spec 73(6):336–342 22005723 10.1159/000327097

[7] Strong MS, Jako GJ (1972) Laser surgery in the larynx. Early clinical experiences with continuous CO2 laser. Ann Otol Rhinol Laryngol 81:791–798 4636137 10.1177/000348947208100606

[8] Eckel HE, Thumfart WF (1992) Laser surgery for the treatment of larynx carcinomas: indications, techniques, and preliminary results. Ann Otol Rhinol Laryngol 101:113–118 1739255 10.1177/000348949210100202

[9] Steiner W, Ambrosch P (2000) Endoscopic laser surgery of the upper aerodigestive tract – with special emphasis on cancer surgery. New York: Georg Thieme

[10] Rudert HH, Werner JA (1995) Endoscopic resections of glottic and supraglottic carcinomas with the CO2 laser. Eur Arch Otorhinolaryngol 252:146 7662348 10.1007/BF00178101

[11] Department of Veterans Affairs Laryngeal Cancer Study Group, Wolf GT, Fisher SG, Hong WK, Hillman R, Spaulding M, Laramore GE, Endicott JW, McClatchey K, Henderson WG (1991) Induction chemotherapy plus radiation compared with surgery plus radiation in patients with advanced laryngeal cancer. N Engl J Med 324(24):1685–90 2034244 10.1056/NEJM199106133242402

[12] Forastiere AA, Goepfert H, Maor M, Pajak TF, Weber R, Morrison W, Glisson B, Trotti A, Ridge JA, Chao C, Peters G, Lee DJ, Leaf A, Ensley J, Cooper J (2003) Concurrent chemotherapy and radiotherapy for organ preservation in advanced laryngeal cancer. N Engl J Med 349(22):2091–2098 14645636 10.1056/NEJMoa031317

[13] Silver CE, Beitler JJ, Shaha AR, Rinaldo A, Ferlito A (2009) Current trends in initial management of laryngeal cancer: the declining use of open surgery. Eur Arch Otorhinolaryngol 266:1333–1352 19597837 10.1007/s00405-009-1028-2

[14] Lallemant B, Chambon G, Garrel R, Kacha S, Rupp D, Galy-Bernadoy C, Chapuis H, Lallemant JG, Pham HT (2013) Transoral robotic surgery for the treatment of T1–T2 carcinoma of the larynx: preliminary study. Laryngoscope 123(10):2485–2490 23918439 10.1002/lary.23994

[15] Kayhan FT, Kaya KH, Sayin I (2012) Transoral robotic cordectomy for early glottic carcinoma. Ann Otol Rhinol Laryngol 121(8):497–502 22953654 10.1177/000348941212100801

[16] Hans S, Baudouin R, Circiu MP, Couineau F, Lisan Q, Crevier-Buchman L, Lechien JR (2022) Open partial laryngectomies: history of laryngeal cancer surgery. J Clin Med 11(18):5352 36142999 10.3390/jcm11185352 PMC9501694

[17] Özkul Y, Ateş D, İmre A, Songu M, Balcı K, Bayrak F, Önal K (2017) Analysis of recurrence after frontolateral laryngectomy. Turk Arch Otorhinolaryngol 55(1):27–30 29392048 10.5152/tao.2017.2080 PMC5782924

[18] Brumund KT, Gutierrez-Fonseca R, Garcia D, Babin E, Hans S, Laccourreye O (2005) Frontolateral vertical partial laryngectomy without tracheotomy for invasive squamous cell carcinoma of the true vocal cord: a 25-year experience. Ann Otol Rhinol Laryngol 114:314–322 15895788 10.1177/000348940511400411

[19] Dedivitis RA, Guimarães AV, Guirado CR (2005) Outcome after partial frontolateral laryngectomy. Int Surg 90(2):113–8 16119718

[20] Mantsopoulos K, Psychogios G, Bohr C, Zenk J, Kapsreiter M, Waldfahrer F, Iro H (2012) Primary surgical treatment of T3 glottic carcinoma: long-term results and decision-making aspects. Laryngoscope 122:2723–2727 22965857 10.1002/lary.23580

[21] Mantsopoulos K, Psychogios G, Koch M, Zenk J, Waldfahrer F, Iro H (2012) Comparison of different surgical approaches in T2 glottic cancer. Head Neck 34:73–77 21374754 10.1002/hed.21687

[22] Knopf A, Ketterer MC, Hoffmann TK, Laban S, Berghaus A, Canis M, Jacobi C, Klussmann JP, Föringer W, Laszig R, Pfeiffer J, Bier H (2024) Treatment regimens for laryngeal and hypopharyngeal squamous cell carcinoma: a real life multicenter study of 2307 patients. Eur Arch Otorhinolaryngol. 10.1007/s00405-024-08990-6 10.1007/s00405-024-08990-6 PMC12680893 39438293

[23] Backes C, Bier H, Knopf A (2017) Therapeutic implications of tumor free margins in head and neck squamous cell carcinoma. Oncotarget 8(48):84320–84328 29137426 10.18632/oncotarget.21035 PMC5663598

[24] Higgins KM, Shah MD, Ogaick MJ, Enepekides D (2009) Treatment of early-stage glottic cancer: meta-analysis comparison of laser excision versus radiotherapy. J Otolaryngol Head Neck Surg 38(6):603–612 19958721

[25] Hartl DM, Ferlito A, Brasnu DF, Langendijk JA, Rinaldo A, Silver CE, Wolf GT (2011) Evidence-based review of treatment options for patients with glottic cancer. Head Neck 33(11):1638–1648 21990228 10.1002/hed.21528

[26] Abdurehim Y, Hua Z, Yasin Y, Xukurhan A, Imam I, Yuqin F (2012) Transoral laser surgery versus radiotherapy: systematic review and meta-analysis for treatment options of T1a glottic cancer. Head Neck 34(1):23–33 21374753 10.1002/hed.21686

[27] Rudert HH, Werner JA, Höft S (1999) Transoral carbon dioxide laser resection of supraglottic carcinoma. Ann Otol Rhinol Laryngol 108(9):819–827 10527270 10.1177/000348949910800901

[28] Ambrosch P (2007) The role of laser microsurgery in the treatment of laryngeal cancer. Curr Opin Otolaryngol Head Neck Surg 15(2):82–8 (**Review**) 17413407 10.1097/MOO.0b013e3280147336

[29] Iro H, Waldfahrer F, Altendorf-Hofmann A, Weidenbecher M, Sauer R, Steiner W (1998) Transoral laser surgery of supraglottic cancer: follow-up of 141 patients. Arch Otolaryngol Head Neck Surg 124(11):1245–1250 9821928 10.1001/archotol.124.11.1245

[30] Steiner W (1993) Results of curative laser microsurgery of laryngeal carcinomas. Am J Otolaryngol 14(2):116–21 8484476 10.1016/0196-0709(93)90050-h

[31] Eckel HE, Schneider C, Jungehülsing M, Damm M, Schröder U, Vössing M (1998) Potential role of transoral laser surgery for larynx carcinoma. Lasers Surg Med 23(2):79–86 9738542 10.1002/(sici)1096-9101(1998)23:2<79::aid-lsm5>3.0.co;2-s

[32] Grant DG, Salassa JR, Hinni ML, Pearson BW, Hayden RE, Perry WC (2007) Transoral laser microsurgery for untreated glottic carcinoma. Otolaryngol Head Neck Surg 137(3):482–486 17765780 10.1016/j.otohns.2007.05.064

[33] Giovanni A, Guelfucci B, Gras R, Yu P, Zanaret M (2001) Partial frontolateral laryngectomy with epiglottic reconstruction for management of early-stage glottic carcinoma. Laryngoscope 111(4 Pt 1):663–668 11359138 10.1097/00005537-200104000-00020

[34] Fiorella R, Di Nicola V, Mangiatordi F, Fiorella ML (1999) Indications for frontolateral laryngectomy and prognostic factors of failure. Eur Arch Otorhinolaryngol 256(8):423–425 10525950 10.1007/s004050050180

[35] Maheshwar AA, Gaffney CC (2001) Radiotherapy for T1 glottic carcinoma: impact of anterior commissure involvement. J Laryngol Otol 115(4):298–301 11276333 10.1258/0022215011907235

[36] Celakovsky P, Kalfert D, Smatanova K, Kordac P, Laco J, Chrobok V (2017) Discordance between clinical and pathological TNM classification: influence on results of treatment and prognosis in patients with laryngeal cancer. Neoplasma 64(2):305–310 28052684 10.4149/neo_2017_219

[37] Bootz F (2020) S3-leitlinie diagnostik, therapie und nachsorge des larynxkarzinoms [Guideline on diagnosis, treatment, and follow-up of laryngeal cancer]. HNO 68(10):757–762 32789706 10.1007/s00106-020-00908-y

